# Supplementary material for: Ectopic Reconstitution of a Spine-Apparatus Like Structure Provides Insight into Mechanisms Underlying Its Formation
Source: bioRxiv. 2024 Apr 16:2024.04.16.589782. Preprint. [Version 1] doi: 10.1101/2024.04.16.589782 (PMC11042382; doi:10.1101/2024.04.16.589782)
Supplement: Supplement 1 [file media-1.pdf]

Supplementary Material

Supplementary Figures

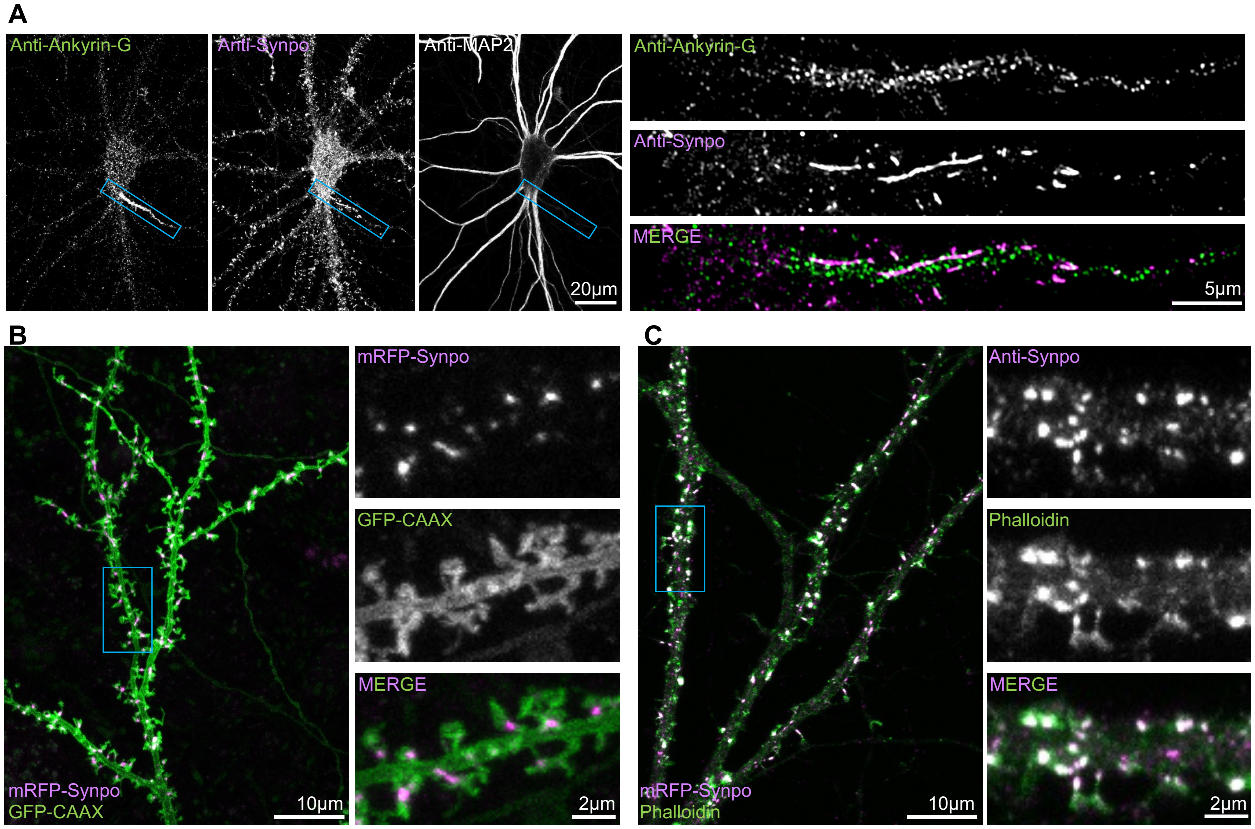

Figure S1. Localization of synaptopodin in dendritic spines and at axonal initial segments. A. In cultured hippocampal neurons, endogenous synaptopodin is detected by immunofluorescence in dendritic spines and at axonal initial segments. Axon initial segment (shown at higher magnification at right) is identified by its presence of immunoreactivity for Ankyrin-G (a marker of this axonal region) and absence of immunoreactivity for the dendritic marker, MAP2. **B.** Co-expression of mRFP-synaptopodin with the plasma membrane marker GFP-CAAX in a cultured hippocampal neuron reveals that synaptopodin puncta are localized at the interface between the neck and the head of dendritic spines, as demonstrated by the high magnification fields shown at right. **C.** Endogenous synaptopodin also colocalizes with a pool of F-actin in dendritic spines. Higher magnification images are shown on the right.

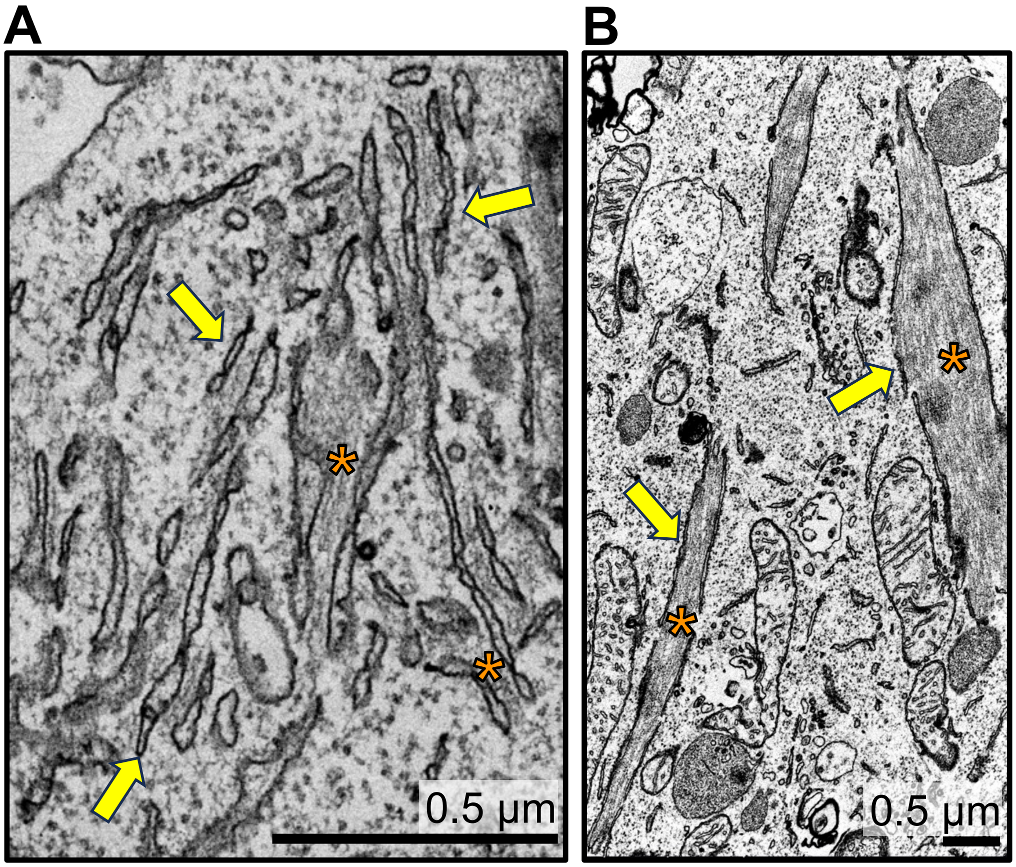

Figure S2. Presence of actin bundles encased by ER in the soma of a cultured hippocampal neuron overexpressing synaptopodin. Note presence of numerous actin bundles of variable thickness encased by ER.

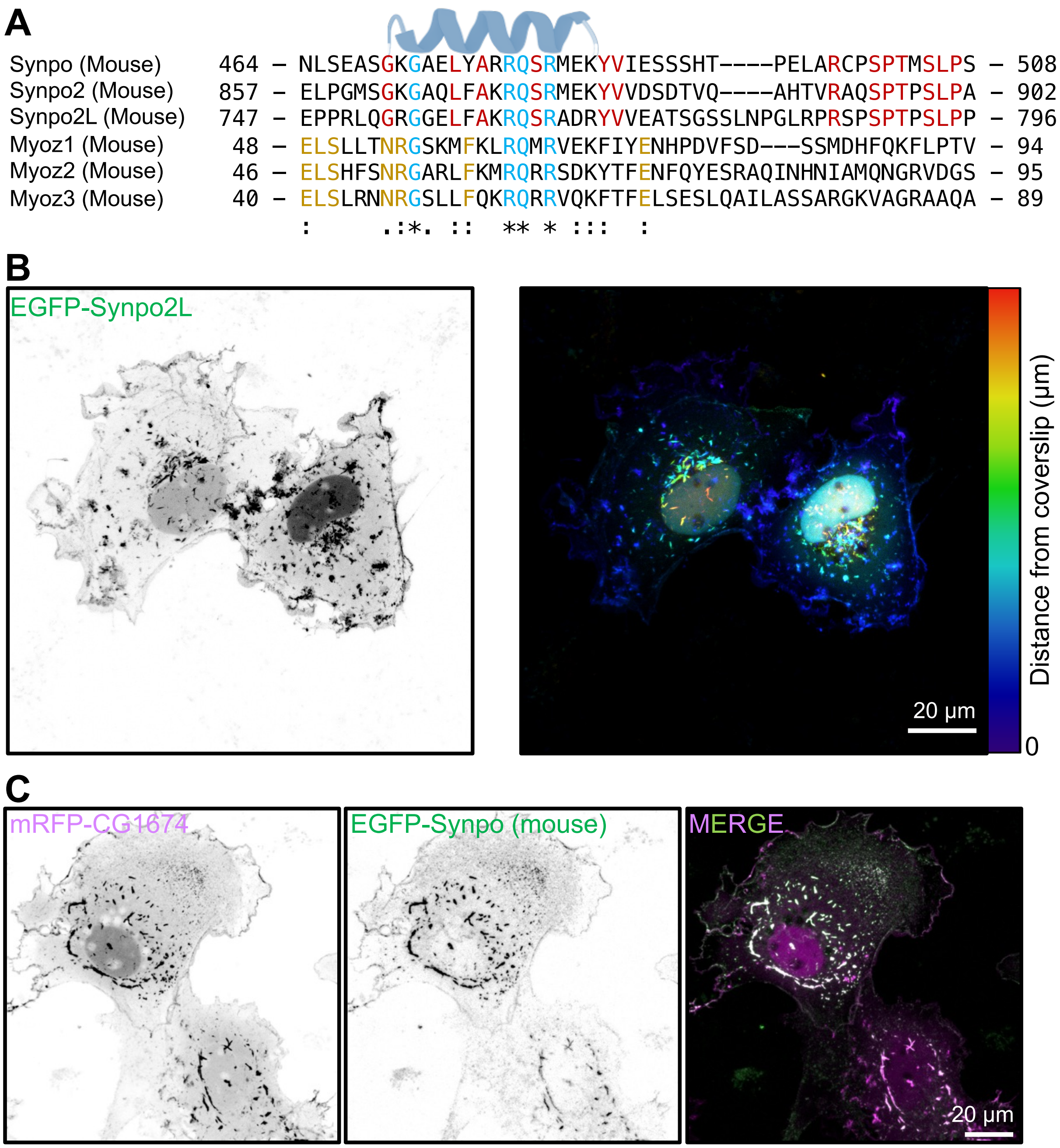

Figure S3. Synaptopodin homologs. A. Conservation of the calsarcin domain in synaptopodin and myozenin mouse paralogues. **B.** COS-7 cell expressing fluorescently tagged Synpo2L. The image on the right is color-coded based on the distance of the synaptopodin fluorescence from the coverslip. Similar to synaptopodin, Synpo2L localizes to plasma membrane in close proximity to the coverslip, and also forms large inclusions that can be farther away from the coverslip. **B.** COS-7 cell co-expressing the fluorescently tagged *Drosophila* orthologue of synaptopodin, mRFP-CG1674, with mouse EGFP-synaptopodin showing precise colocalization of the two proteins.

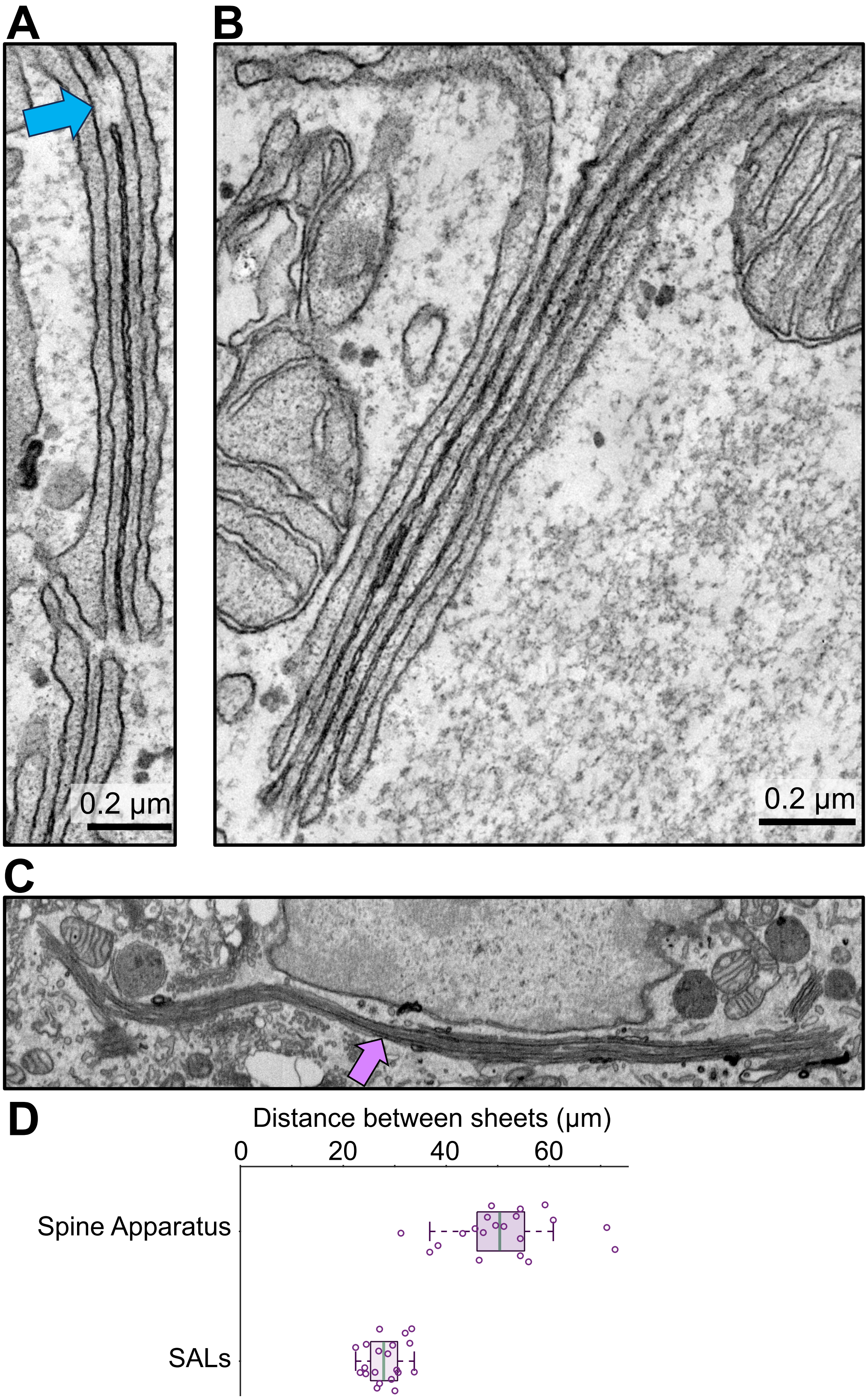

Figure S4. Generation of SALs in COS-7 cells. A-B. Examples of SALs induced by expressing synaptopodin-ER. A blue arrow shows a fenestration in one of the sheets. **C.** A single plane image

from a stack of FIB-SEM images of a COS-7 cell expressing synaptopodin-ER. A very long SAL is indicated by a magenta arrow (see also Video 2). **D**. Quantification of the distances between ER sheets in the spine apparatus of dendritic spines and in SALs. Each circle represents the distance measurement between two opposed ER sheets in a different stack of a spine apparatus or a SAL.

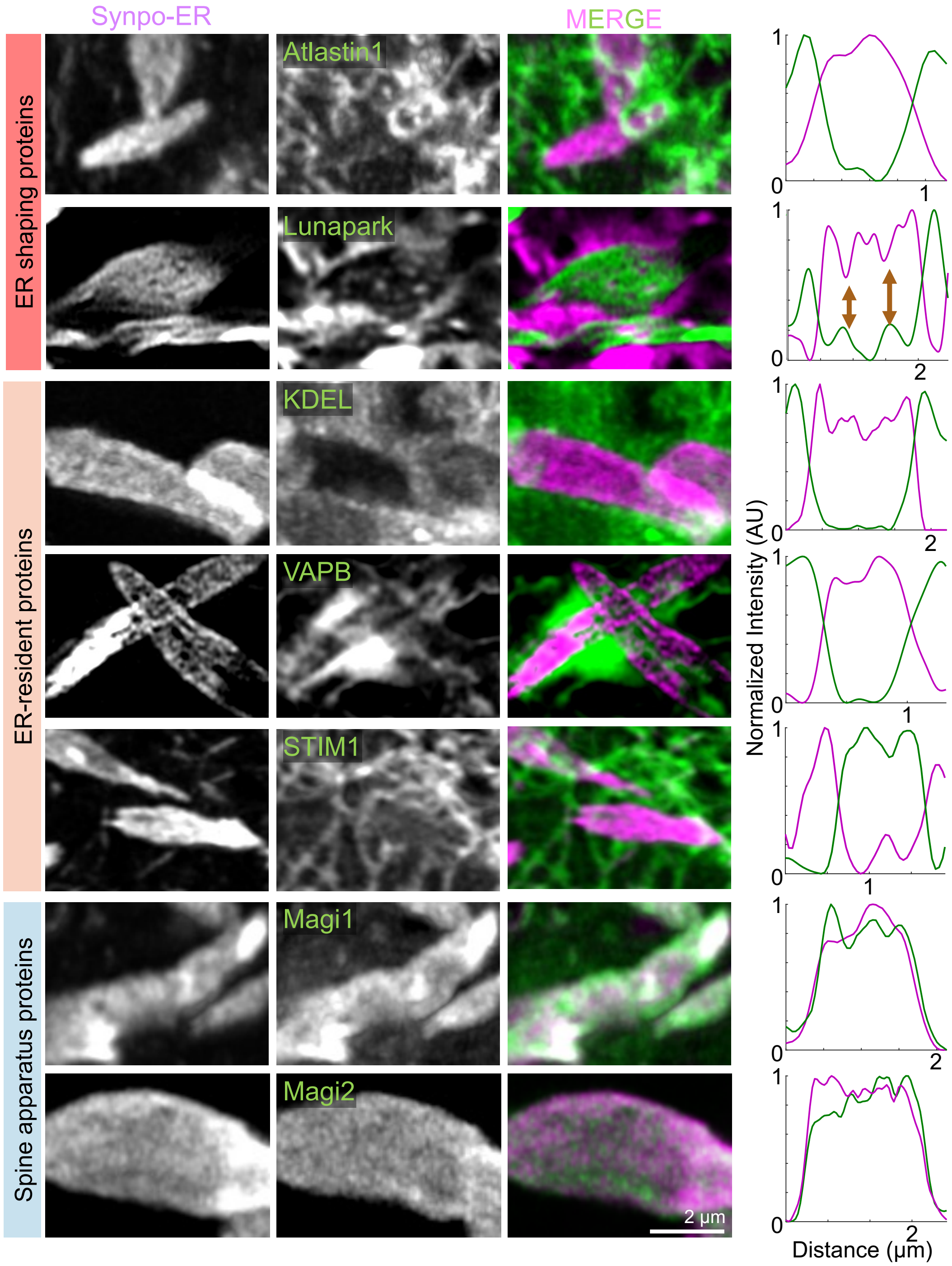

Figure S5. Molecular characterization of SALs. A. AiryScan images of SALs where the localization of fluorescent Synpo-ER is compared (in pairwise comparisons) to the localization of co-expressed fluorescently tagged ER shaping proteins, other ER housekeeping proteins and spine apparatus proteins. Line plots are shown on the right. The coincidence of the florescence signal of Lunapark-mCherry within the sheets of SALs, alongside discontinuities in Synpo-ER fluorescence, is indicated by gold arrows in the line plot.

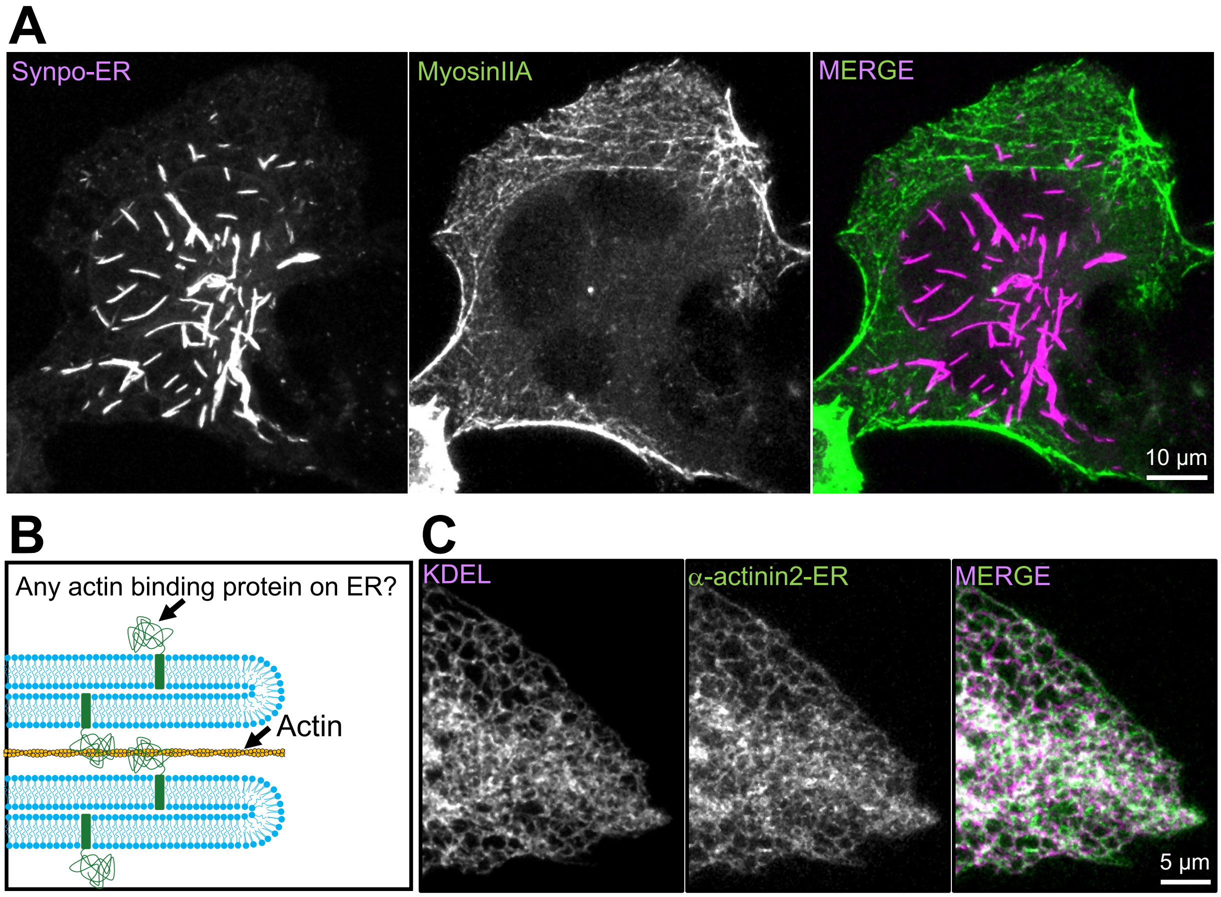

Figure S6. Insufficiency of actin binding for induction of SAL formation. A. COS-7 cell coexpressing Synpo-ER with MyosinIIA-GFP. **B.** Schematic of a model proposing that targeting any actin-binding protein to the ER would result in the expansion and stacking of ER sheets forming SALs. However, this model is disproven (**C**), as the targeting α-actinin2 to ER by fusing it to Sec61β does not induce SAL formation in COS-7 cells.

Supplementary tables

Table S1. List of antibodies.

Protein (epitope)	Company; Catalog number	Antibody species	Working dilution for immunocytochemistry	Working dilution for immunoblotting
Synaptopodin	Sigma S9442-200UL	Rabit	1:1000	1:2500
Ankyrin-G	NeuroMab 75-147 Clone 106/65	Mouse	1:500	N/A
MAP2	Invitrogen PA1-10005	Chicken	1:100	N/A
Phalloidin-Alexa Fluor 488	Thermofisher A12379		1:300	NA

Table S2. List of cloning methods and reagents.

Construct	Cloning method	Primers	Backbone	Template/ insert
pAAV-HA-EGFP-Synaptopodin	Digestion / ligation with AgeI and ClaI	N/A	pAAV-BioID2-Synaptopodin	pAAV-HA-EGFP-Pdlim7
mRFP-FKBP12-Synaptopodin	Digestion / In-Fusion	Primer 1: ACCGGCGCCttgtacaccggactcagatctcgaagc<br>Primer 2: agtccggacttgtacaattccagttttagaagctccacatc	mRFP-Synaptopodin	mRFP-FKBP12
mRFP-Synaptopodin Δ(384-473)	In-Fusion	Primer 1: tgaccccggagctctatgcccgccgc<br>Primer 2: agagctccggggtcaccttgggcttctcc	N/A	mRFP-Synaptopodin
mRFP-FKBP12-Synaptopodin (1-383)	In-Fusion	Primer 1: TGACCCCGTAACACACCGCGGGCCCG<br>Primer 2: TGTGTTACGGGGTCACCTTGGGCTTCTCC	N/A	mRFP-FKBP12-Synaptopodin
mRFP-FKBP12-Synaptopodin (474-690)	In-Fusion	Primer 1: TCGACTTCGAGCTCTATGCCCGCCGC<br>Primer 2: AGAGCTCGAAGTCGACTGCAGAATTCGAAGC	N/A	mRFP-FKBP12-Synaptopodin
mRFP-FKBP12-Synaptopodin ΔCalsarcin	In-Fusion	Primer 1: CCAATCAGTCCTGGAAGTACACCACTAACGC<br>Primer 2: TCCAGGACTGATTGGGTTTGGGCTTCGG	N/A	mRFP-FKBP12-Synaptopodin
mRFP-FKBP12-Synaptopodin (380-690)	In-Fusion	Primer 1: tcgacttcGTGACCCCGAATCCAGATTTGC<br>Primer 2: GGGTCACGAAGTCGACTGCAGAATTCGAAGC	N/A	mRFP-FKBP12-Synaptopodin
mRFP-FKBP12-Synaptopodin (380-690) ΔCalsarcin	In-Fusion	Primer 1: CCAATCAGTCCTGGAAGTACACCACTAACGC<br>Primer 2: TCCAGGACTGATTGGGTTTGGGCTTCGG	N/A	mRFP-FKBP12-Synaptopodin(380-690)
mRFP-CG1674	In-Fusion	For CG1674:<br>Primer 1:<br>GCAGTCGACTTCATGGATTCTACTTTAAATATTGAGAATG<br>Primer 2:<br>GCCCGCGGTGTGTTAAAAATCAGAGTACGGTAGATTTC<br>For Backbone:<br>Primer 1: TAACACACCGCGGGCCCG<br>Primer 2: CATGAAGTCGACTGCAGAATTCGA	mRFP-Synaptopodin	From IP15312
mRFP-Synaptopodin 2L	In-Fusion	For Synaptopodin2L:<br>Primer 1:<br>GCAGTCGACTTCATGGGTGCTGAGGAGGAGGTGC<br>Primer 2:<br>GCCCGCGGTGTGTTACAACTGGTGCCCTGCCCC<br>For Backbone:<br>Primer 1: TAACACACCGCGGGCCCG<br>Primer 2: CATGAAGTCGACTGCAGAATTCGA	mRFP-Synaptopodin	DNASU: HsCD00861847
α-actinin-2-AcGFP-Sec61β	In-Fusion	Primer 1:<br>CGCTAGCGCTACCGGATGAACCAGATAGAGCCCGGC<br>Primer 2:<br>CATGGTGGCGACCGGTAGATCGCTCTCCCCGTAGAG	AcGFP-Sec61β	pEGFP-α-actinin-2
